# Supplementary material for: Reconciling Mining with the Conservation of Cave Biodiversity: A Quantitative Baseline to Help Establish Conservation Priorities
Source: PLoS One. 2016 Dec 20;11(12):e0168348. doi: 10.1371/journal.pone.0168348 (PMC5173368; doi:10.1371/journal.pone.0168348)
Supplement: S1 Dataset — (ZIP) [file pone.0168348.s002.zip › Taxa/Serra Norte/SN_2013/N4E/Lista N4E-10.pdf]

CAVIDADE N4E-0010

Classe	Ordem	Fam/Outros	Gên/Outros	Espécie	S-U	U-S	Zon.
Annelida	Oligochaeta			jovem		1	A
Arachnida	Acari	(Oribatida)		jovem	X		
Arachnida	Acari	(Parasitengonina)		sp.13		X	A
Arachnida	Acari	(Parasitengonina)		sp.2		X	P
Arachnida	Acari	Ixodidae	*Amblyomma*	sp.1	X		
Arachnida	Acari	Mesostigmata		jovem	X		
Arachnida	Acari	Prostigmata		jovem	X		
Arachnida	Amblypygi	Charinidae	*Charinus*	sp.1	X	1	A
Arachnida	Amblypygi	Phrynidae	*Heterophrynus*	*longicornis*	X		
Arachnida	Araneae	Barychelidae		jovem		2	P
Arachnida	Araneae	Ctenidae		jovem	1	1	P
Arachnida	Araneae	Drymusidae	*Drymusa*	jovem	X		
Arachnida	Araneae	Ochyroceratidae	*Ochyrocera*	sp.	X		
Arachnida	Araneae	Ochyroceratidae	*Ochyrocera*	sp.1		X	P/A
Arachnida	Araneae	Ochyroceratidae	*Ochyrocera*	sp.3		X	P
Arachnida	Araneae	Ochyroceratidae	*Speocera*	sp.1	X	X	P/A
Arachnida	Araneae	Pholcidae		jovem	X		
Arachnida	Araneae	Theraphosidae		sp.	X		
Arachnida	Araneae	Theridiidae		jovem		X	P
Arachnida	Araneae	Theridiosomatidae	*Plato*	sp.1	X	X	P/A
Arachnida	Opiliones	Escadabiidae		sp.2	X	X	A
Arachnida	Palpigradi	Eukoeneniidae	*Allokoenenia*	sp.1	X	X	A
Arachnida	Pseudoscorpiones	Chernetidae	*Spelaeochernes*	sp.1	X	X	P/A
Chilopoda	Scutigeromorpha	Pselliodidae	*Sphendononema*	*guildingii*	X		
Diplopoda	Polydesmida	Chelodesmidae		jovem	X	2	A
Diplopoda	Polydesmida	Fuhrmannodesmidae		jovem		X	A
Diplopoda	Polydesmida	Pyrgodesmidae		sp.1	X	X	P
Diplopoda	Spirostreptida	Pseudonannolenidae	*Pseudonannolene*	jovem		2	A
Entognatha	Collembola	Cyphoderidae		sp.1		X	P/A
Entognatha	Collembola	Isotomidae		sp.1		X	P/A
Entognatha	Collembola	Paronellidae		sp.1		X	P
Entognatha	Collembola	Paronellidae		sp.4		X	A
Entognatha	Collembola	Sminthuroidea		sp.2		X	A
Entognatha	Diplura	Campodeidae		sp.1	X	X	P
Entognatha	Diplura	Japygidae		sp.1		X	A
Insecta	Blattodea			jovem	X	2	A
Insecta	Coleoptera	Carabidae	*Coarazuphium*	sp.n.1	X	X	A
Insecta	Coleoptera	Carabidae	*Coarazuphium*	sp.n.2	X		A
Insecta	Coleoptera	Curculionidae	Scolytinae	sp.	X		
Insecta	Coleoptera	Dytiscidae	*Copelatus*	*cessaima*	X	X	A
Insecta	Coleoptera	Staphylinidae	Pselaphinae	sp.12	X		
Insecta	Coleoptera	Staphylinidae	Pselaphinae	sp.35		X	A
Insecta	Coleoptera	Tenebrionidae		jovem	X		
Insecta	Diptera	Culicidae	*Culex*	sp.	X	X	A
Insecta	Diptera	Drosophilidae	*Drosophila*	sp.	X		
Insecta	Diptera	Mycetophilidae		sp.	X		
Insecta	Diptera	Phoridae	Metopininae	sp.	X	X	A
Insecta	Diptera	Psychodidae	Phlebotominae	sp.		X	P
Insecta	Heteroptera	Cydnidae		jovem	X	X	A
Insecta	Heteroptera	Reduviidae	Emesinae	sp.		1	P
Insecta	Heteroptera	Reduviidae	Reduviinae	sp.		1	P
Insecta	Homoptera	Cixiidae		sp.3	X	X	A
Insecta	Hymenoptera	Formicidae	*Camponotus*	sp.1	X	X	P/A
Insecta	Hymenoptera	Formicidae	*Dolichoderus*	aff. *bispinosus*		X	P
Insecta	Hymenoptera	Formicidae	*Gnamptogenys*	sp.1		X	P

Insecta	Hymenoptera	Formicidae	*Ochetomyrmex*	sp.1		X	A
Insecta	Hymenoptera	Formicidae	*Pachycondyla*	sp.2		X	P
Insecta	Hymenoptera	Formicidae	*Pseudomyrmex*	aff. *opaciceps*		X	A
Insecta	Isoptera			sp.		X	P
Insecta	Lepidoptera	Roeslerstammiidae		sp.		X	P
Insecta	Lepidoptera	Tineoidea		jovem	X		
Insecta	Neuroptera	Myrmeleontidae		jovem		X	P
Insecta	Orthoptera	Phalangopsidae	*Paraclodes*	sp.	X		
Insecta	Orthoptera	Phalangopsidae	*Phalangopsis*	sp.	X		
Insecta	Psocoptera	Psyllipsocidae		jovem		X	P
Gastropoda	Pulmonata	Bulimulidae	*Naesiotus*	sp.		X	A
Gastropoda	Pulmonata	Subulinidae	*Leptinaria*	sp.	X		
Gastropoda	Pulmonata	Systrophiidae	*Happia*	sp.	X	X	A
Malacostraca	Isopoda	Armadillidae		sp.1	X		
Malacostraca	Isopoda	Scleropactidae	*Circoniscus*	*buckupi*	X		
Amphibia	Anura			sp.	1	3	P
Mammalia	Chiroptera	Emballonuridae	*Peropteryx*	sp.	3		E/P
Mammalia	Chiroptera	Phyllostomidae	*Carollia*	*perspicillata*		1	A
